# A Hybrid CNN–LSTM Algorithm for Online Defect Recognition of CO_2_ Welding

**DOI:** 10.3390/s18124369

**Published:** 2018-12-10

**Authors:** Tianyuan Liu, Jinsong Bao, Junliang Wang, Yiming Zhang

**Affiliations:** 1 College of Mechanical Engineering, Dong Hua University, Shanghai 201620, China; tyliu@mail.dhu.edu.com (T.L.); junliangwang@dhu.edu.cn (J.W.); 2 College of Literature, Science and the Arts, The University of Michigan, Ann Arbor, MI 48109, USA; yimingz@umich.edu

**Keywords:** deep learning, CNN, LSTM, CO_2_ welding, molten pool, online monitoring

## Abstract

At present, realizing high-quality automatic welding through online monitoring is a research focus in engineering applications. In this paper, a CNN–LSTM algorithm is proposed, which combines the advantages of convolutional neural networks (CNNs) and long short-term memory networks (LSTMs). The CNN–LSTM algorithm establishes a shallow CNN to extract the primary features of the molten pool image. Then the feature tensor extracted by the CNN is transformed into the feature matrix. Finally, the rows of the feature matrix are fed into the LSTM network for feature fusion. This process realizes the implicit mapping from molten pool images to welding defects. The test results on the self-made molten pool image dataset show that CNN contributes to the overall feasibility of the CNN–LSTM algorithm and LSTM network is the most superior in the feature hybrid stage. The algorithm converges at 300 epochs and the accuracy of defects detection in CO_2_ welding molten pool is 94%. The processing time of a single image is 0.067 ms, which fully meets the real-time monitoring requirement based on molten pool image. The experimental results on the MNIST and FashionMNIST datasets show that the algorithm is universal and can be used for similar image recognition and classification tasks.

## 1. Introduction

Welding is a dynamic, interactive, and non-linear process. The monitoring of welding defects is a difficult problem due to these characteristics of welding. The main difficulties in this task include deciding when a defect occurred and which type of defect occurred. In the actual welding process, skilled welders can dynamically adjust the welding process from observing the state of molten pool to prevent welding defects. That gave rise to our idea that we could adjust the welding process by observing the molten pool. An accurate mapping model between the molten pool image and the weld quality is a vital part of this method [1,2]. The molten pool images are independently used as inputs to this model. A typical molten pool image contains many objects, such as welding wire, arc, molten pool, weld seam, metal accumulation, splash, smoke, etc. Although the molten pool is the main part of the whole image, it is necessary to consider all objects and the relationship between various objects for the purpose of accurately reflecting the welding information through the molten pool image. Therefore, extracting the features from all objects in the molten pool image and hybridizing the different features are the key to establishing an accurate mapping model.

The research of molten pool image can be divided into two categories. One of them is based on multiple molten pool images. The main idea is to find the mutation rule of molten pool characteristic signals when welding defects occur by multi-level (time domain, frequency domain) statistical analysis of the characteristics of multiple molten pool images [3,4,5,6,7,8]. This idea can synthetically consider the molten pool images’ information of the whole welding process, and the features used for analysis are generally primary features, which are relatively easy to design and obtain. However, this idea can only be used to analyze the overall welding quality and locate the welding defects after welding is complete and does not satisfy requirements of real-time monitoring of welding. Another idea is based on single molten pool images, which is more suitable for an online monitoring process [9,10,11,12,13]. In studying molten pool images of the welding process, the most original method is to manually design and identify the statistics of the characteristics of molten pool (length, width, area, spatter number, etc.) and then identify the molten pool state. Although this method is highly interpretable, it requires a lot of prior knowledge and is very time-consuming. Furthermore, such a model poorly adapts to other image classification problems. With the development of deep learning, convolutional neural network (CNN) replaced the process of human design and the extraction of primary features, achieving great results [14,15,16]. However, for the purpose of further improving the accuracy of defect recognition, more convolutional layers need to be stacked in the feature fusion stage, which will bring huge computational cost, making real-time monitoring infeasible. In view of the existing problems in the feature hybrid stage, principal component analysis (PCA) and fully connected layers are widely used to combine the results of feature extraction [17,18,19,20,21]. Although PCA has great interpretability, such a deterministic process may leave out features with small contributions, and these features may entail important information about welding quality. Therefore, the process of feature information fusion lacks the ability of intelligent fusion. Adding a fully connected layer and adjusting the weight of each shallow feature using back propagation can play a certain role in intelligent hybrid of features; however, this hybrid method is often too simple and insufficient to extract high-level abstract information.

Traditional neural networks (including CNN) assume that all inputs and outputs are independent of each other, while the basic assumption of recurrent neural network (RNN) is that there is an interaction between the input sequences, and this feature of RNN provides a new approach to feature hybrid [22,23]. References [24,25,26,27,28,29,30] propose a method for intelligently hybridizing the features of each individual in the input sequence using the long short-term memory network (LSTM, a variant of RNN), which can extract the long-term dependencies of the data features in the sequence to improve the recognition accuracy. However, the original input of the whole online molten pool status recognition task is a single molten pool image at a certain moment rather than a sequence of images. Therefore, in view of the above problems and the complexity of the molten pool images, this paper proposes an innovative strategy. In the feature extraction stage, multiple convolutional kernels are used to scan the whole molten pool image to obtain the redundant features of all objects in the molten pool image. Due to the distance that the convolution kernel slides each time is less than the size of the convolution kernel itself, and there are overlapping parts in each scan area of the convolution kernel, so the feature blocks extracted by the convolution kernel also depend strongly on each other. When describing a thing, we often hope to construct a set of bases, which can form a complete description of a thing. The same is true in the same level of a convolution network, that is, the relationship between feature maps extracted from the same level of convolution kernels lies in the formation of a description of images on different bases at the same level. So in the stage of feature fusion, several feature images extracted by CNN are unified and reconstructed into a two-dimensional feature matrix that contains the correlation information from the interior of a single feature image and from multiple feature images. In order to improve the accuracy of molten pool image recognition, each row of the feature matrix is considered as a basic unit to be hybridized, and the number of rows is considered as the length of a sequence. In this way, the single image of molten pool is converted into “sequential” data in this sense. Then, the long-term dependencies property of the LSTM network is used to filter and fusion the rows of the feature matrix to obtain high-level abstract information. In this case, the model is transformed into a multi-input single-output model like text sentiment analysis. Each input can be understood as a contribution of the feature vector at this time step to the overall molten pool image identification task in the context. The CNN–LSTM algorithm proposed in this paper establishes the end-to-end mapping relationship between molten pool image and welding defects. The advantage of this algorithm is that it can intelligently learn the best hybrid features through the error back propagation algorithm in the shallow CNN network for a single molten pool image to meet the engineering requirements for real-time monitoring of the welding process. In this paper, the molten pool image is obtained by a CO_2_ welding test. The feasibility, superiority to other models, and contribution sources of the proposed algorithm are tested and studied. The experiment is carried out on the MNIST and FashionMNIST datasets to illustrate the versatility of the CNN–LSTM algorithm. The feature hybrid method in this paper also has certain reference significance for similar image recognition tasks.

## 2. Deep Learning Model Based on CNN–LSTM

**Figure 1:**
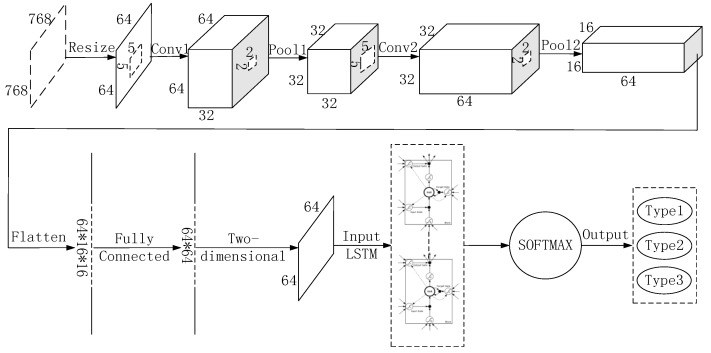
Convolutional neural network and long short-term memory network (CNN–LSTM) algorithm overall architecture.

A CNN is a neural network that uses convolution operation instead of traditional matrix multiplication in at least one layer of the network. It is especially used to deal with data with similar grid structures, a data structure common in computer vision and image processing [14]. The 2D image data can be directly used as the bottom-level input of a CNN, and then the essential features of the image are extracted layer-by-layer through convolution and pooling operations. These features have the invariance of translation, rotation, and scaling. However, the output layer of the traditional CNN is fully connected with the hidden layer. This feature fusion method which takes all outputs of the convolutional layer is far too simple for the purpose of our model. Problems with this method include bad kernels, multiple kernels extracting the same information, and unnecessary information extracted by kernels. It is possible to extract deeper image features and improve recognition accuracy by increasing the number of convolutional kernels, convolutional layers, and pooling layers. But it will undoubtedly lead to a huge network, thereby increasing the cost of computation, and also facing the risk of overfitting [14,15]. As a time recurrent neural network, LSTM is suitable for processing the sequence problem with time dependence. The input feature tensor is selectively forgotten, input and output through three threshold structures. It can filter and fuse the empty input, similar information, and unnecessary information extracted by the convolutional kernels, so that the effective feature information can be stored in the state cell for a long time. Therefore, an algorithm combining CNN and LSTM was proposed in literature [24,25,26,27,28,29,30], which has achieved good results in gesture recognition, voice recognition, rainfall prediction, machine health condition prediction, text analysis, and other fields. However, the above literature is targeted at prediction tasks, and the input of LSTM is also a batch of images in time series. But the molten pool online monitoring process is faced with the identification task. The original input of this task is a single molten pool image taken by the camera at a certain moment. The ideas of sequence dependency are clearly inapplicable to this problem. Therefore, in view of the above problems, this paper proposes an algorithm named CNN–LSTM for the online monitoring task of the molten pool, which hybridizes the advantages of CNN and LSTM. The overall architecture of CNN–LSTM is shown in Figure 1.

**Figure 2:**
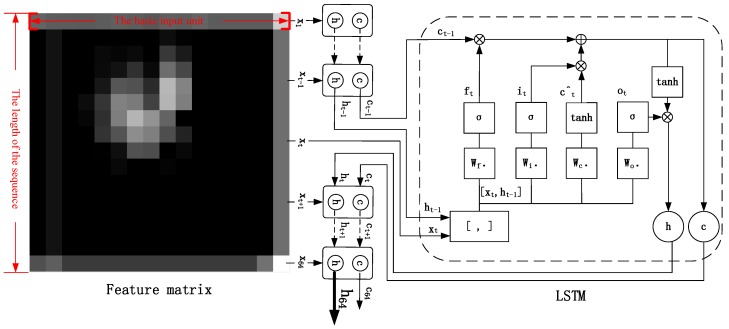
Feature hybrid mechanism of CNN-LSTM.

The CNN–LSTM algorithm is designed for the recognition task of a single image. Since CNN’s feature extraction is adaptive and self-learning, our model can overcome the reliance of feature extraction and data reconstruction relying on human experience and subjective consciousness in traditional recognition algorithms. It uses multiple convolutional kernels to scan the entire molten pool image to obtain redundant features of all objects as candidates. In the feature hybrid stage, the three-dimensional feature tensor output from the last layer of CNN is firstly stretched into a one-dimensional feature vector. As mentioned earlier, this vector has all feature information extracted by convolutional kernels, which includes some blank information, similar information, unnecessary information, and so on. Then the feature vector is mapped to two-dimensional space as the input of LSTM. Each row of the feature matrix is considered as a basic unit to be hybridized. Each time step reads a row of feature information and divides a feature matrix into several time steps to read. In this way, the single image of molten pool is converted into “sequential” data in this sense. The LSTM network is used to extract the dependencies between each row of feature matrix, so as to filter and hybridize the features extracted from the CNN network. Figure 2 shows the innovation of the CNN–LSTM network. In the time interval of the CNN–LSTM network identification molten pool image, the input of LSTM network at time *t* includes the output *h_t_*_−1_ and unit state *c_t_*_−1_ at time *t* − 1, and the network’s input *x_t_* of current time. The feature tensor and the cell state can be filtered and hybridized by three carefully designed threshold structures, so that the effective features extracted from the CNN can be stored in cell state for a long time and the invalid features are forgotten.

## 3. Model Implementation and Parameter Details

### 3.1. Model Implementation Process

It can be seen from Figure 1 that this algorithm is mainly divided into feature extraction stage based on CNN and feature fusion stage based on LSTM. In the feature extraction stage, the forward propagation process of the image signal is as follows: it is assumed that the *l* layer is a convolutional layer, and the *l* − 1 layer is a pooling layer or an input layer. Then the calculation formula of the *l* layer is:

(1) $$x_{j}^{l}=f\left(\sum_{i\in M_{j}}x_{i}^{l-1}\times k_{ij}^{l}+b_{j}^{l}\right)$$

The $x_{j}^{l}$ on the left of the above equation represents the *j*th feature image of the *l* layer. The right side shows the convolution operation and summation for all associated feature maps $x_{i}^{l-1}$ of the *l* − 1 layer and the *j*th convolutional kernel of the *l*th layer, and then adds an offset parameter, and finally passes the activation function *f*(*). Among them, *l* is the number of layers, *f* is the activation function, *M_j_* is an input feature map of the upper layer, *b* is offset, and *k* is convolutional kernel.

Assuming that the *l* layer is pooling layer (down sampling layer), the *l* − 1 layer is the convolutional layer. The formula for the *l* layer is as follows:

(2) $$x_{j}^{l}=f\left(\beta_{j}^{l}down\left(x_{j}^{l-1}\right)+b_{j}^{l}\right)$$

In the above formula, *l* is the number of pooling layer, *f* is the activation function, down(*) is the down sampling function; *β* is the down sampling coefficient, and *b* is the offset.

In the feature hybrid stage, the network uses three threshold structures to control the state of the cell that preserves long-term memory. The meaning of long short-term memory is: *c_t_* corresponds to long-term memory, and ${\tilde{c}}_{t}$ corresponds to short-term memory. The σ(*) in Expressions (3), (4), and (7) is a Sigmoid function. If the output of Sigmoid function is 1, then the information is fully remembered. If the output is 0, then it is completely forgotten. If the output is the value between 0 and 1, it is the proportion of information to be remembered. The gate is actually equivalent to a fully connected layer and its input is a vector and output is a real vector between 0 and 1. It uses the output vector of the “gate” multiplied by the vector we want to control. The forgetting gate *f_t_* determines how much historical information can be remained in a long-term state *c_t_*; ${\tilde{c}}_{t}$ is used to describe the short-term state of current input. The input gate *i_t_* determines how much of the current network input information can be added to the long-term state *c_t_*; the output gate *o_t_* controls how much of the aggregated information is available as the current output. The expressions are as follows:

(3) $$f_{t}=\sigma \left(W_{f}•\left[h_{t-1},x_{t}\right]+b_{f}\right),$$

(4) $$i_{t}=\sigma \left(W_{i}•\left[h_{t-1},x_{t}\right]+b_{i}\right),$$

(5) $${\tilde{c}}_{t}=tanh(W_{c}•\left[h_{t-1},x_{t}\right]+b_{c}),$$

(6) $$c_{t}=f_{t}\circ c_{t-1}+i_{t}\circ {\tilde{c}}_{t},$$

(7) $$o_{t}=\sigma \left(W_{o}•\left[h_{t-1},x_{t}\right]+b_{o}\right),$$

(8) $$h_{t}=o_{t}\circ tanh(c_{t}),$$

The above are the formulas of the forward propagation process of the image signal. “•” means matrix multiplication, and “∘” means multiplication by elements of the same position. The output of the last time step of the LSTM network includes current unit state *c_64_* and current output *h_64_*. We take *h_64_* as the overall output of the LSTM part, which is the input of SOFTMAX. After the signal passed through the SOFTMAX, the judgment of the category is given in the form of probability. In the algorithm training stage, the network adopts the error back propagation method to iteratively update the weights and offsets until the number of epochs is reached.

### 3.2. Model Parameter Details

Tensorflow is a deep learning framework developed by Google. It provides a visual tool Tensorboard that can display the learning process of algorithms. In order to realize the CNN–LSTM algorithm proposed in this paper, the relevant hyper-parameters under this deep learning framework are set as follows: in view of the fact that the gray image of the molten pool taken by the charge coupled device (CCD) camera is too large (768 × 768), it brings great difficulty to the network operation. Therefore, the gray image size is first converted to 64 × 64. In the first convolutional layer (Conv1), there are 32 convolutional kernels with a size of 5 × 5. The convolution stride is 1, and the padding method is same to ensure that the image size is unchanged after convolution. At this time, the image data is converted to 64 × 64 × 32. The first pooling layer (Pool1) uses the maximum pooling. The pooling window with a size of 2 × 2, the pooling stride is 1, and the padding method is same. At this time, the image data is converted to 32 × 32 × 32. In the second convolution layer (Conv2), there are 64 convolution kernels with a size of 5 × 5. The convolution stride is 1, and the padding method is same. At this time, the image data is converted to 32 × 32 × 64. The second pooling layer (Pool2) uses the maximum pooling. The pooling window with a size of 2 × 2, the pooling stride is 1, and the padding method is same. At this time, the image data is converted to 16 × 16 × 64. The fully connected layer adjusts the feature matrix to 64 × 64, and each time step takes one row as the input to the LSTM network. There are 64 time steps in the total. There are 100 hidden units in the LSTM network. Finally, the classification results of defects are obtained through SOFTMAX. In addition, the learning rate of this network is set to 10^−4^, and Adam is chosen as the optimizer.

**Figure 3:**
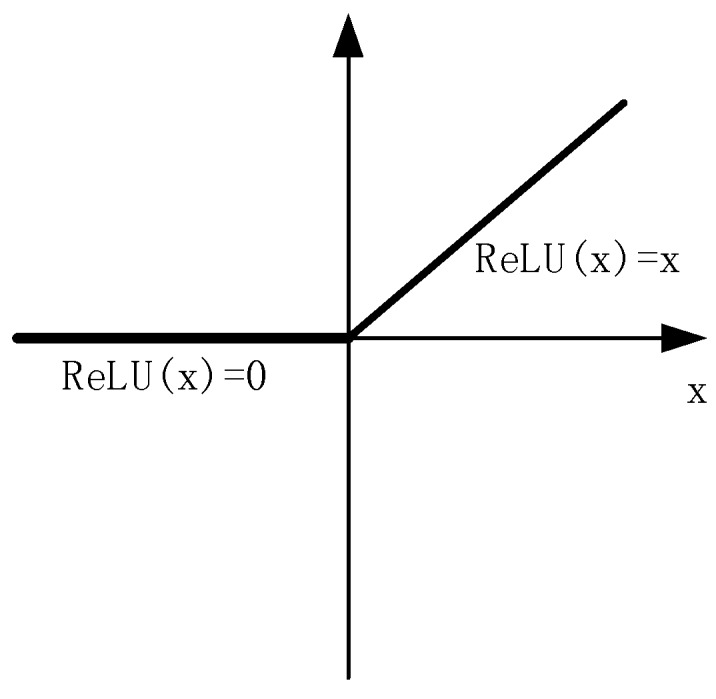
Schematic diagram of the ReLU function.

**Figure 4:**
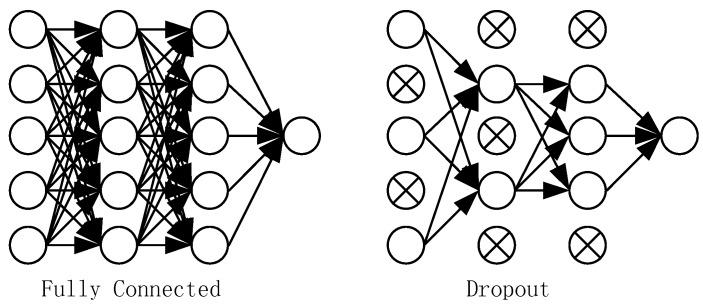
Schematic diagram of the random Dropout method.

Considering the small sample size, in order to prevent overfitting and reduce the amount of calculation, the first layer convolution result, the second layer convolution result and the fully connected layer result all use ReLU (Rectified Linear Units) activation function: ReLU(x) = max(0,x), which is shown in Figure 3. The ReLU activation function is more expressive than the linear function. The convergence rate of ReLU is faster than that of nonlinear activation functions such as Sigmoid and Tanh. Moreover, since the derivative of ReLU activation function is equal to 1, it can help with vanishing gradient problem [31,32]. In order to further reduce the possibility of overfitting caused by the small sample size, a random dropout method is used in the fully connected layer which is shown in Figure 4. Some neurons are stochastically deactivated at each epoch. Dropout decreases the dependencies between nodes and reduce overfitting by turning the CNN into an ensemble classifier of many weak classifiers. The dropout parameter is set to 0.5 in our model.

## 4. Test Design and Environment

### 4.1. Test Design

First of all, in order to help understand the mechanism of feature extraction and evolution of the algorithm, the operation results of each convolution and pool layer will be visualized. Secondly, in order to show the feasibility and generalization ability of CNN–LSTM algorithm, the training performance and testing performance will be compared. The size of the original image was converted into 32 × 32, 64 × 64, and 128 × 128 as the initial input. The contribution source of the feasibility of the algorithm was illustrated by the influence of different input sizes on the composition algorithm. Among the tasks related to image feature extraction, CNN has been widely proved to be superior to traditional algorithms. Therefore, in order to fully reflect the superiority of the algorithm and illustrate the contribution sources of the superiority, performance comparison tests were conducted under the same hyper-parameters with the composition algorithm (CNN, LSTM) and CNN-3 (add a convolution and pooling layer, respectively). Finally, in order to illustrate the versatility of the CNN–LSTM algorithm, the performance of the algorithm was tested on the MNIST and FashionMNIST datasets in Appendix A.

In addition, in order to guarantee the fairness of the comparison test, other hyper-parameters such as convolution kernel size, pool window size, stride, network learning rate, activation function, optimizer, dropout value, LSTM’s hidden layer unit number, etc., were set to be same. The algorithm was analyzed and compared using three criteria: recognition accuracy, convergence speed, and recognition time.

### 4.2. Test Environment

**Figure 5:**
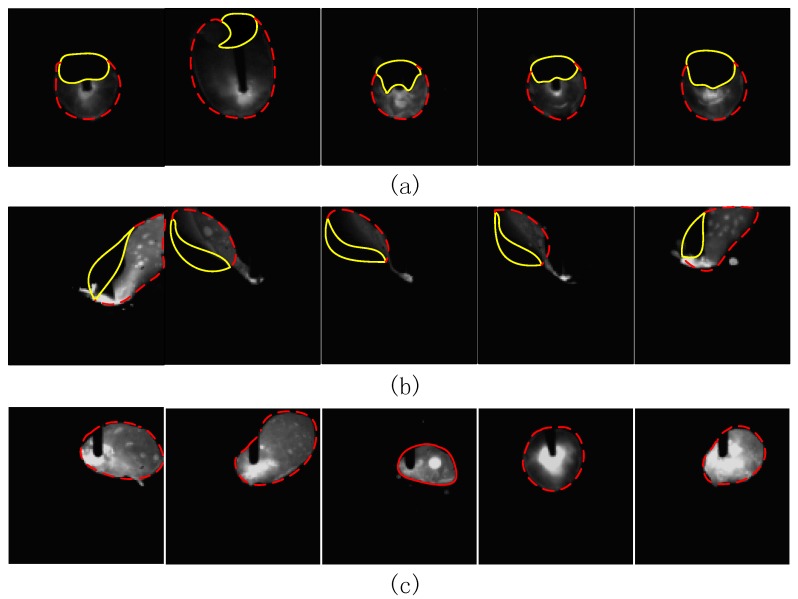
Part of the sample set images. (**a**) welding through; (**b**) welding deviation; (**c**) normal welding.

In terms of data sources, this paper relies on the key laboratory of Robotics and Welding Technology of Guilin University of Aerospace Technology to carry out the CO_2_ welding test. In the actual welding process, welding defects are caused by a variety of factors and have great uncertainty. Through pre-processing, a total of 500 molten pool images of the three most common types of welding including welding through, welding deviation, and normal welding were collected. The original size of the images were 768 × 768. There were 300 pictures per class in the training set and 100 pictures in each class in the validation set and testing set. The tail of the molten pool corresponding to the welding through defect will leak to the back of the base metal and appear as a shadow on the image (Figure 5a, yellow area). Welding through defects are mainly caused by the welding current being too large, welding speed being too slow, the base material too thin, the base material not uniform, and so on. The weld pool corresponding to the weld deviation defect will deviate from the predetermined weld seam. Welding deviation defects are mainly caused by the vibration of the walking mechanism, the low accuracy of the positioning, the instability of the arc, and so on. At this point, the molten pool will deviate from the predetermined weld, which is reflected in the image as a part missing from the molten pool (Figure 5b, yellow area). A normal molten pool has an elliptical shape. Figure 5 shows a partial picture of the sample set.

The algorithm performs performance tests under the ubuntu16.04 operating system, a GTX1080Ti graphics card, a hardware environment of 64 GB running, and the Tensorflow deep learning framework.

## 5. Test Results and Analysis

### 5.1. Visual Analysis

**Figure 6:**
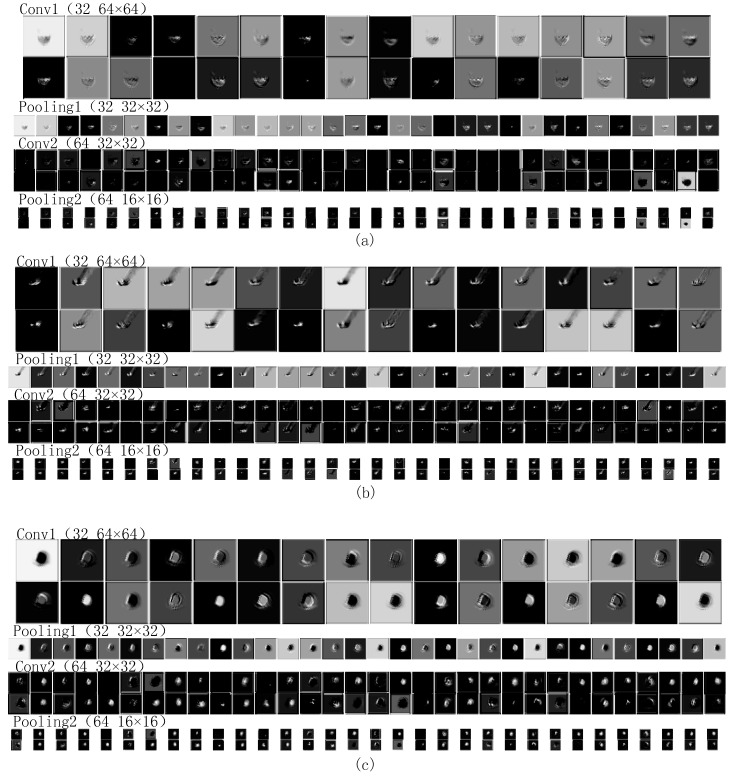
The feature images of the molten pool in three states: (**a**) welding through; (**b**) welding deviation; (**c**) normal welding.

According to the details of the network framework, the algorithm has two layers of convolution and two layers of pooling for feature adaptive extraction of the molten pool image. Figure 6 shows the feature extraction and evolution mechanism of CNN.

As a whole, the comparison between the convolution results of the first layer and the convolution results of the second layer shows that the activation degree of the background of the molten pool decreases with the deepening of the convolution layer. The attention of convolution kernels gradually concentrates on the characteristic information of molten pool and ignores the background. The convolution kernels in the first layer are mainly used to detect low-order features, such as the edge, angle, and curve of the molten pool. The convolution kernels in the second layer are mainly used to detect the combined features of low-order features, such as the arc and shape of the molten pool. The result of the first convolution layer has a high spatial resolution, which is conducive to the accurate positioning of the target, such as the separation of the molten pool and the background but lacks robust feature representation. The spatial resolution of the second-layer convolution result is reduced due to the pooling operation, resulting in weaker positioning functions, but with deeper abstract features, and thus distortion tolerance [14,15]. The results of the second layer convolution are more ambiguous than the first layer, but the unique part of the category is highlighted. The maximum pooling operation can reduce the parameter calculation amount to prevent overfitting while better retaining the main texture features of the molten pool. It can be seen that maximum pooling of the first layer has a partial enhancement to the features extracted by the first convolution layer. However, after the maximum pooling of the second layer, some relatively abstract discrete blocks appeared, which are difficult to see by the naked eye. In addition, a small number of feature images are black or very similar to other feature images, which means that the convolution kernels failed to extract information or similar information were extracted by multiple convolution kernels. Therefore, it is necessary to select and combine the feature information by using LSTM network before the classification.

Specifically, in the same convolution layer, the convolution kernels extract different features in different molten pool states. Due to the excessive energy density of the weld, the molten pool will collapse in the middle, resulting in shadow behind the molten pool image (Figure 5a, yellow area). This feature is an irregular feature map similar to the elliptical gap in the convolution results. The welding deviation defects caused by improper groove angle, uneven assembly clearance or low precision of welding robot, resulting in the molten pool is only half (Figure 5b, yellow area), its own irregularity is very strong, resulting in a large deviation and irregularity of convolution results. Due to the regular shape of the molten pool in the normal state (Figure 5c, red area), the feature images extracted from the convolution kernels are also approximately elliptic, and the molten pool in the normal state is brighter than the other two states, so the convolution kernels have also extracted more bright features. In the same molten pool state, taking the normal state as an example, as mentioned above: the first layer of convolution kernels are more concerned with the characteristics of the approximate elliptical edge of the molten pool; the second layer of convolution kernels focuses on the overall morphological features of the approximate ellipse of the molten pool; the pooling layer improves computational efficiency while preserving texture features that can represent grayscale distributions of pixels and surrounding spatial neighborhoods.

### 5.2. Feasibility Analysis

**Figure 7:**
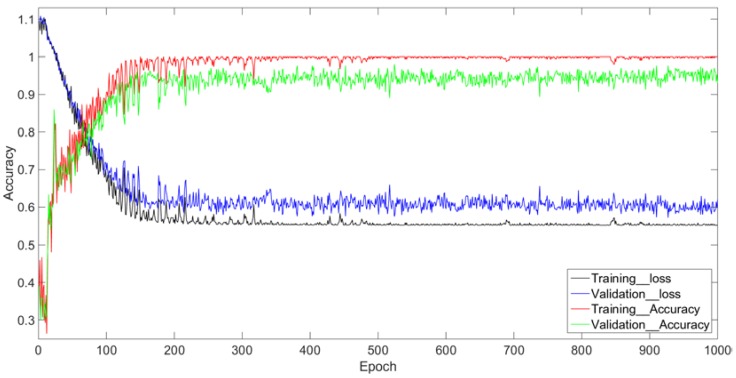
The recognition accuracy of this network training and testing process.

The loss curve of CNN–LSTM algorithm in the training process is shown in Figure 7. On the training set, the CNN–LSTM network begins to converge after about 200 epochs and the convergence process is stable. On the validation set, CNN–LSTM begins to converge after about 300 epochs. Although the convergence process fluctuates slightly, the overall trend of loss is clearly decreasing. Although the accuracy of CNN–LSTM on the validation set is slightly lower than that on the training set, with the increase of the training epoch, the accuracy on the verification set does not decrease, and the recognition accuracy on the test set is 94%. Through the above analysis, we can find that the algorithm of maximum pooling, ReLU activation function, and random dropout can effectively suppress the overfitting due to the sparse set of samples, thus achieving excellent performance on both the training and testing stage, which embodies the effectiveness and strong generalization ability of the algorithm.

**Figure 8:**
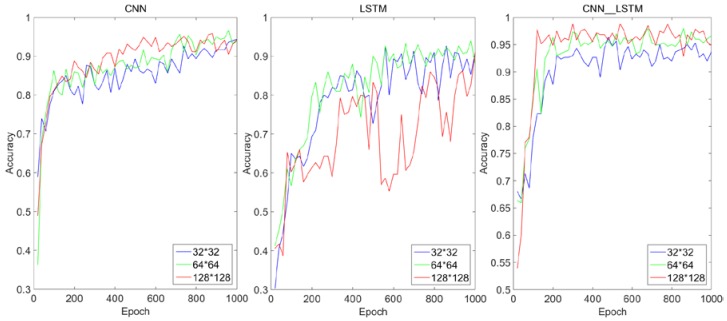
Influence of different input sizes on each algorithm.

The effect of different input sizes on each algorithm is shown in Figure 8. It can be seen that with the increase of input sequence size, the convergence speed and recognition accuracy of the CNN network increase significantly. This is because with the increase of input sequence size, the details and features of molten pool image are more abundant, and the strong feature adaptive extraction ability of CNN network comes into play. But for the LSTM algorithm, the recognition accuracy is the highest when the size of the input image is 64 × 64. When the input size is 128 × 128, the recognition accuracy is lower than that input with smaller size. This is likely because that when the size of the input sequence is small, the details and features of the molten pool image are small and not obvious, and LSTM cannot extract too much effective characteristic sequence information of the molten pool. When the input sequence is long, LSTM can extract more effective sequence information, and at the same time, it can exert its strong gradient retention and long-term dependence ability. But when faced with a particularly long sequence, because the traditional neural network model uses an encoder-decoder structure, the model of this structure usually encodes the input sequence into a fixed-length vector representation. For short input sequences, the model can learn a reasonable vector representation. However, the problem with this model is that when the input sequence is very long, it is difficult for the model to learn a reasonable vector representation, so it is difficult to retain all necessary information [33,34,35]. However, the CNN–LSTM network proposed in this paper increases the convergence speed and recognition accuracy as the input sequence size increases. This indicates that the method of automatic feature extraction by CNN has the largest overall contribution to the feasibility of the CNN–LSTM algorithm, and the method of intelligent fusion of feature tensor by LSTM’s long term dependence has the second largest contribution to the feasibility of the CNN–LSTM algorithm.

### 5.3. Performance Analysis

**Figure 9:**
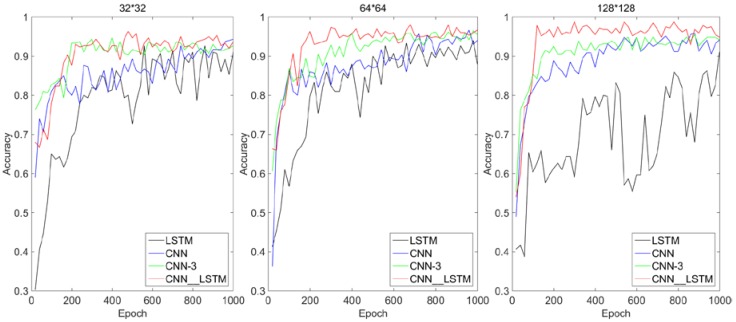
Performance comparison of three algorithms under different input dimensions.

The training performance comparison between the CNN–LSTM algorithm and each component algorithm under the same hyper-parameter is shown in Figure 9. Taking the input image size 64 × 64 as an example, in terms of convergence speed, the LSTM network starts to converge from about 700 epochs and eventually converges to about 90% accuracy. The CNN network converges from about 600 epochs and eventually converges to about 93% accuracy. The CNN-3 network converges from about 400 epochs and eventually converges to about 94% accuracy. The CNN–LSTM algorithm starts to converge from about 300 epochs and eventually converges to about 96% accuracy. In terms of defect recognition accuracy on the test set, the recognition accuracy of LSTM network is 88%, the recognition accuracy of CNN network is 89%, the recognition accuracy of CNN-3 network is 91%, the recognition accuracy of CNN–LSTM network is 94%. In terms of defect recognition speed, it can be found from Table 1 that LSTM algorithm has the fastest recognition speed under any input image size, followed by CNN. The CNN–LSTM has more training parameters due to the hybrid of CNN and LSTM, so it takes more time, but it is still faster than the CNN-3 which contains the three-layer convolution and pooling. In addition, when the input image size is 128 × 128, although CNN-3 can get a recognition accuracy of 94% on the test set, the recognition time of a single image is five times longer than that of the CNN–LSTM network when the input image size is 64 × 64. In the process of online monitoring of the molten pool state, the most important thing is to ensure the recognition accuracy of the algorithm, followed by the recognition time of a single image. Therefore, the CNN–LSTM network can guarantee high-recognition accuracy by adjusting the input size in the shallow network under the requirement of high-frequency molten pool monitoring while CNN needs to stack more convolution layers to improve the recognition accuracy, but it undoubtedly brings huge real-time computational cost. Therefore, considering all algorithms’ recognition accuracy, convergence speed and single image recognition speed comprehensively, the CNN–LSTM algorithm is superior to all the other competitors in real-time welding applications. This superiority is a result of using LSTM in the feature fusion stage, which filters and hybridizes the feature tensor extracted by CNN with rows as the unit. This method can consume a shorter recognition time with the guarantee of reaching a very high accuracy.

## 6. Discussion

In order to meet the engineering requirements of high accuracy and real time in welding in an online monitoring process, a CNN–LSTM algorithm was proposed based on the traditional deep learning method. The original input of the model is a single image, and the LSTM network processes the feature map extracted by CNN instead of the original sequence, as in the literature. The motivation for using LSTM in this paper was to intelligently fuse the feature information that CNN has extracted, rather than to extract the dependencies between each individual in the sequence. The feasibility of the algorithm is based on using multiple convolution kernels to scan the whole image to obtain the redundant features of the molten pool. The hybrid algorithm was designed in such a way that the rows of the feature matrix extracted by CNN are considered as the basic units and put into the LSTM network for a feature hybrid. The algorithm has high accuracy and short time to identify defects in the molten pool, which completely meets the need of online monitoring in the molten pool. The experiment on the self-made molten pool image dataset shows that the contribution of the feasibility of the algorithm is more derived from the CNN’s feature adaptive extraction capability. However, the superiority of the algorithm is derived from using LSTM in the feature hybrid stage, which filtered and hybridized the feature tensor extracted by CNN in rows. The successful application of the CNN–LSTM algorithm on the MNIST and FashionMNIST datasets show that the motivation of this algorithm is universal when dealing with similar non-strict sequential image data.

Although the feature hybrid method in the CNN–LSTM algorithm is superior to the traditional methods, there are still some shortcomings. In future research work, we should first consider obtaining more defect types and sample sets of molten pool. Secondly, the choice of hyper-parameters should be fully studied in the process of network construction. Thirdly, welding quality should be used as a bridge to establish a corresponding model between the welding process and the weld pool defects. Finally, a feedback control model should be established between the monitoring results of the molten pool and the welding process to realize online monitoring of the welding process based on the molten pool.

## Figures and Tables

**Table 1:** The recognition accuracy and recognition time of different algorithms under different input sizes.

	Algorithm Type	Input Size		
		32 × 32	64 × 64	128 × 128
**Accuracy (Recognition Time (t/ms))**	**LSTM**	0.85	0.88	0.8
		(0.017)	(0.033)	(0.167)
	**CNN**	0.88	0.89	0.92
		(0.02)	(0.06)	(0.233)
	**CNN-3**	0.90	0.91	0.94
		(0.04)	(0.099)	(0.33)
	**CNN–LSTM**	0.92	0.94	0.95
		(0.033)	(0.067)	(0.2667)

**Figure A1:**
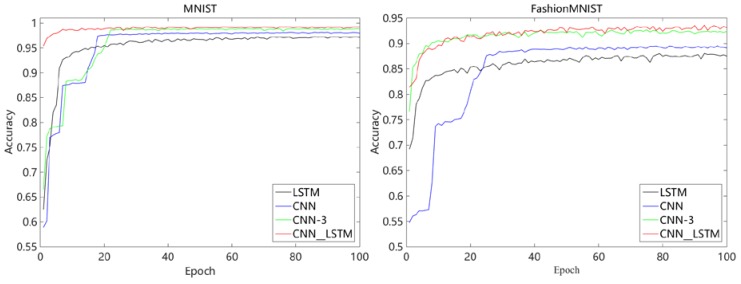
The representation of different algorithms on MNIST and FashionMNIST datasets.

## Appendix A

The motivation of the CNN–LSTM algorithm is that each row of the feature matrix is regarded as a basic input unit of the LSTM network, and the number of rows of the feature matrix is regarded as the length of the sequence, so that the feature matrix can be filtered and hybridized by using the long-term dependence ability of the LSTM network. In order to illustrate the universality of the CNN–LSTM algorithm, this algorithm was tested on MNIST and FashionMNIST datasets which have no strict timing dependence and then compared with basic algorithms. The MNIST dataset includes 10 categories of handwritten numerals from 0 to 9, and FashionMNIST includes 10 categories such as T-shirt, Coat, Sandal, Bag, etc. The original size of the images in both datasets is 28 × 28. Figure A1 shows the variation of accuracy of different algorithms on these two public datasets during training. It can be seen that on the relatively simple dataset of MNIST, all four algorithms are very stable during training. Although the improvement accuracy of CNN–LSTM is not obvious compared with that of CNN-3, the CNN–LSTM algorithm converges faster. Both CNN-3 and CNN–LSTM converge rapidly on the more complex dataset of FashionMNIST, but with the increase of training epoch, CNN-3 is unable to improve the accuracy, and the accuracy of CNN–LSTM algorithm is still rising. It can also be seen from Figure A1 that CNN–LSTM has a higher accuracy than other algorithms in the early stages of training, which shows that the CNN–LSTM algorithm can quickly adapt to different image scenarios. Table A1 shows the recognition accuracy of each algorithm on the test set and the recognition time of a single image. It can be seen that the CNN–LSTM algorithm takes less time than the CNN-3 algorithm with similar recognition accuracy. As mentioned earlier, although the feature extraction ability of CNN is very strong, the long-term dependency ability of LSTM network is stronger than that of stacked convolution layer for fusing ability of feature tensor extracted by CNN. The successful application of the CNN–LSTM algorithm on MNIST and FashionMNIST datasets show that our innovative model of combining CNN and LSTM is generic when dealing with similar non-strict sequential image data.

**Table A1:** The recognition accuracy of each algorithm on the test sets and the recognition time of a single image.

	Algorithm	Dataset	
		MNIST	FashionMNIST
**Accuracy (Recognition time (t/ms))**	**LSTM**	0.9797	0.8671
		(0.008)	(0.01)
	**CNN**	0.9911	0.8885
		(0.01)	(0.012)
	**CNN-3**	0.992	0.9122
		(0.016)	(0.018)
	**CNN-LSTM**	0.9922	0.9128
		(0.014)	(0.016)
